# Neural network aided flexible joint optimization with design of experiment method for nuclear power plant inspection robot

**DOI:** 10.3389/fnbot.2023.1049922

**Published:** 2023-02-08

**Authors:** Gang Wang, Jiawei Li, Xinmeng Ma, Xi Chen, Jixin Wang, Songjie Han, Biye Pan, Ruxiao Tian

**Affiliations:** ^1^Science and Technology on Underwater Vehicle Laboratory, Harbin Engineering University, Harbin, China; ^2^College of Mechanical and Electrical Engineering, Heilongjiang Institute of Technology, Harbin, China; ^3^College of Information and Communication Engineering, Harbin Engineering University, Harbin, China

**Keywords:** flexible joint, inspection robot, neural network, optimization, topology

## Abstract

**Introduction:**

The flexible joint is a crucial component for the inspection robot to flexible interaction with nuclear power facilities. This paper proposed a neural network aided flexible joint structure optimization method with the Design of Experiment (DOE) method for the nuclear power plant inspection robot.

**Methods:**

With this method, the joint's dual-spiral flexible coupler was optimized regarding the minimum mean square error of the stiffness. The optimal flexible coupler was demonstrated and tested. The neural network method can be used for the modeling of the parameterized flexible coupler with regard to the geometrical parameters as well as the load on the base of the DOE result.

**Results:**

With the aid of the neural network model of the stiffness, the dual-spiral flexible coupler structure can be fully optimized to a target stiffness, 450 Nm/rad in this case, and a given error level, 0.3% in the current case, with regard to the different loads. The optimal coupler is fabricated with wire electrical discharge machining (EDM) and tested.

**Discussion:**

The experimental results demonstrate that the load and angular displacement keep a good linear relationship in the given load range and this optimization method can be used as an effective method and tool in the joint design process.

## 1. Introduction

Nuclear power plays a great role in promoting energy transition, and the safety, maintenance, and overhaul of nuclear power facilities are crucial links in the nuclear power industry (Larsen and Babineau, 2020; Liu et al., 2020; Mallants et al., 2020; Park and Lee, 2020; Zhao et al., 2020). In recent years, more and more robots have been developed to serve nuclear power plants (Kim et al., 2021).

Kim et al. (2014) designed a laser-guided underwater robot for reactor vessel inspection. Li et al. (2019) introduced a remote robot solution for maintenance of diverter in European demonstration power plant (DEMO) fusion reactor. The kinematic design of the robot has been optimized for the DEMO access, and inverse kinematics of the robot solution are introduced. Bird et al. (2022) presented the Vega robot, a small, low-cost, potentially disposable ground robot designed for nuclear decommissioning. Vega has been establishment and demonstrated to many other organizations in the UK nuclear industry, including Sellafield Ltd, intending to move to active deployments in the future. Sayed et al. (2022) presented a survey of current robotic systems that can operate in such extreme environments and offer a novel approach to solving the challenges they impose, encapsulated by the mission statement of providing structure in unstructured environments and exemplified by a new self-assembling modular robotic system, the Connect-R.

With the development of robotics, the rigid joint of robots can not meet the special requirements in some environments, and flexible joints for robots got more and more researchers' attention (Hogan, 2022). Currently, the flexible joint involves in the techniques of spring, rubber, pneumatic muscle, function materials, etc. Jutinico et al. (2017) address the dependence of the impedance control performance on the force control and proposed the Markovian control approach that improves the force control robustness. Lee and Oh (2019) developed a robot leg driven by the Series Elastic Actuator (SEA) with biarticular coordination to effectively transmit actuator torque to the operational space. Zhang et al. (2020) proposed a novel cable-driven rotary series elastic actuator (SEA) to implement remote actuation and verified the performance of both the torque and impedance controllers in simulation and experiments. Cao et al. (2020) proposed a specialized ankle joint muscle reflex control algorithm for human upright standing push-recovery.

The spring-type structure dominates the flexible joint applications, which use the spring to couple the motor and load. The flexible joints can overcome the disadvantages of the rigid joints and improve the robot's stability in the environment of impact and vibration (Van Ham et al., 2009). Lagoda et al. (2010) developed an electric serial elastic actuated joint for robotic gait rehabilitation training, which utilized a dual-spiral plane spring to couple the motor and output, successfully realizing the flexible drive of the joint. Chaichaowarat et al. (2020) proposed a dual-spiral type plane spring to connect the gearbox output shaft and load shaft end, which successfully realized a flexible drive for rotation. Dos Santos et al. (2017) designed an active knee orthosis driven by a rotary serial elastic actuator. In the actuator, a plane flexible coupler with nine petals was used to connect the gearbox shaft and load shaft with a 200 Nm/rad stiffness and tested.

High-precision parametric design of the flexible coupler is the key to improving the performance of flexible joints. Carpino et al. (2012) proposed a torsional spring for a serial elastic actuator and arranged it in parallel to acquire enough torque load capacity for robot joints and verified the design. Paine et al. (2015) proposed a distributed torque control design and provided suppression of disturbance according to the joint order and torque error controlled to a level of 1.38%. Palli et al. (2011) proposed a design method of a nonlinear flexible part for a compact design actuator. Negrello et al. (2015) developed a novel serial flexible element and applied it to humanoid robots, and successfully improved the impact resistance of the robot. Baccelliere et al. (2017) designed a modularized flexible drive device for the robot arms and validated the impact resistance with experiments. Sun et al. (2018a,b) proposed an Archimedes spiral repositioning mechanism for a variable stiffness structure and further improved the output structure to enlarge the stiffness range. Generally, the spring or flexible element can be parameterized, and the finite element method can be used to analyze the performance, such as stiffness, but the parameter optimization is still a big challenge because of the complex geometry.

This paper proposed a neural network aided flexible joint structure optimization method with Design of Experiment (DOE) for the nuclear power plant inspection robot. With this method, the dual-spiral flexible coupler of the joint was optimized with regard to the minimum mean square error of the stiffness successfully. The optimal flexible coupler was demonstrated and tested. The remaining article is structured as follows. In Section 2, the inspection robot and flexible joint solutions are proposed, and the response relationship between each parameter and the stiffness of the flexible coupler is studied. In Section 3, the topological structure parameters of the flexible coupler are optimized by the neural network and the DOE optimization method. In Section 4, the performance evaluation experiment conducted is shown and the abilities achieved by the flexible joint are presented, along with the performance indicators. Finally, in Section 5, the conclusions of this article are presented.

## 2. Methods and materials

According to the work requirements (flexible interaction between robots and nuclear power facilities), an inspection robot and flexible joint solutions are proposed. The topology structure of a flexible coupler is proposed and represented by parameterization, and the processing materials of the flexible coupler are selected. The finite-element method (FEM) was used to analyze the response relationship between each parameter and stiffness.

### 2.1. The flexible joint of inspection robot

**Figure 1:**
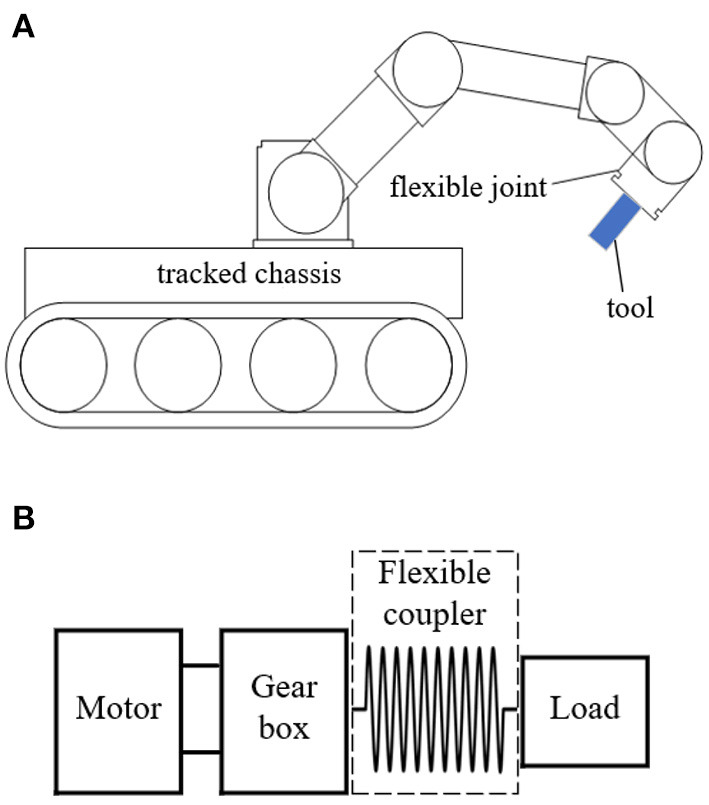
The schematic diagram of the inspection robot and the flexible joint. **(A)** Schematic diagram of the inspection robot. **(B)** Schematic diagram of the flexible joint.

The schematic diagram of the inspection robot composed of a machine arm (with six flexible joints) and tracked chassis is shown in Figure 1. The tool equipped at the end of the arm can move in six degrees of freedom with the support of six flexible joints. Every joint is driven by an independent motor through the gearbox, and the output of the gearbox is connected with the external load by the flexible coupler. The flexible coupler can avoid/filter out the impact forces in operation and ensure the force feedback stable and reliable.

### 2.2. The topological design parameters and materials of the flexible joint

**Figure 2:**
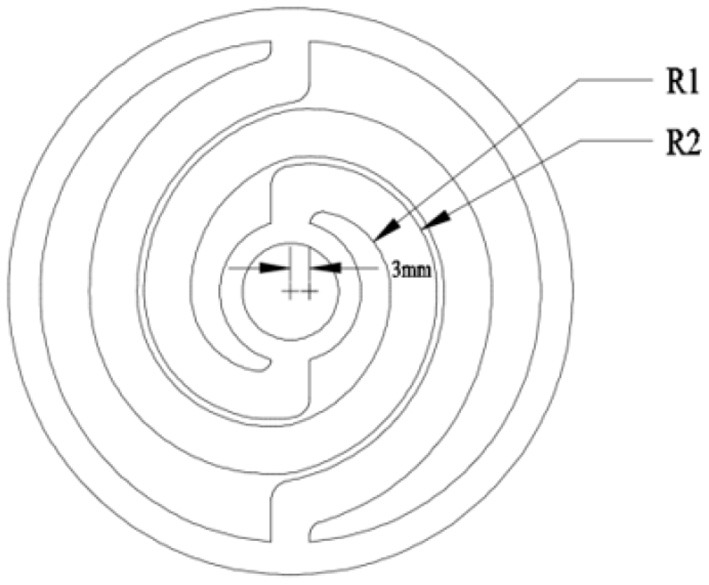
Symmetric dual-spiral flexible coupler.

The typical symmetric dual-spiral structure is selected for the flexible coupler, as shown in Figure 2. The topological design can be parameterized as the initial inner radium, *R*_1_, initial outer radium, *R*_2_, and beam thickness, *h*, with a center offset of 3 mm. The spiral beam width is constant and equals *R*_2_*-R*_1_. The spiral starts from 0° and ends at 360°. If the initial radius, *R*_1_ and *R*_2_, and beam thickness, *h*, are given, the geometry of the flexible coupler can be created. The three structural parameters of the flexible coupler are involved in the geometrical optimization.

The available spring steel specifications are listed in Table 1.

**Table 1:** Available spring steels for the flexible coupler.

**Material**	**65 Mn**	**60 CrMnBA**	**55 CrMnA**	**50 CrVA**
Yield strength, MPa	430	1080	1080	1127

Comparing the available spring steels, 50 CrVA is the best with regard of the yield strength, can be used as material for flexible coupler. The mechanical properties of 50 CrVA are listed in Table 2.

**Table 2:** Mechanical property of the spring steel, 50CrVA.

**Property**	**Value**
Yield strength	≥1,127 MPa
Tension strength	≥1274 MPa
Hardness	≤321 HB
Density	7.85 g/cm^3^
Young's modulus	2.06 × 10^5^ MPa
Poison's ratio	0.3

### 2.3. The effects of the geometrical parameters on the stiffness

**Figure 3:**
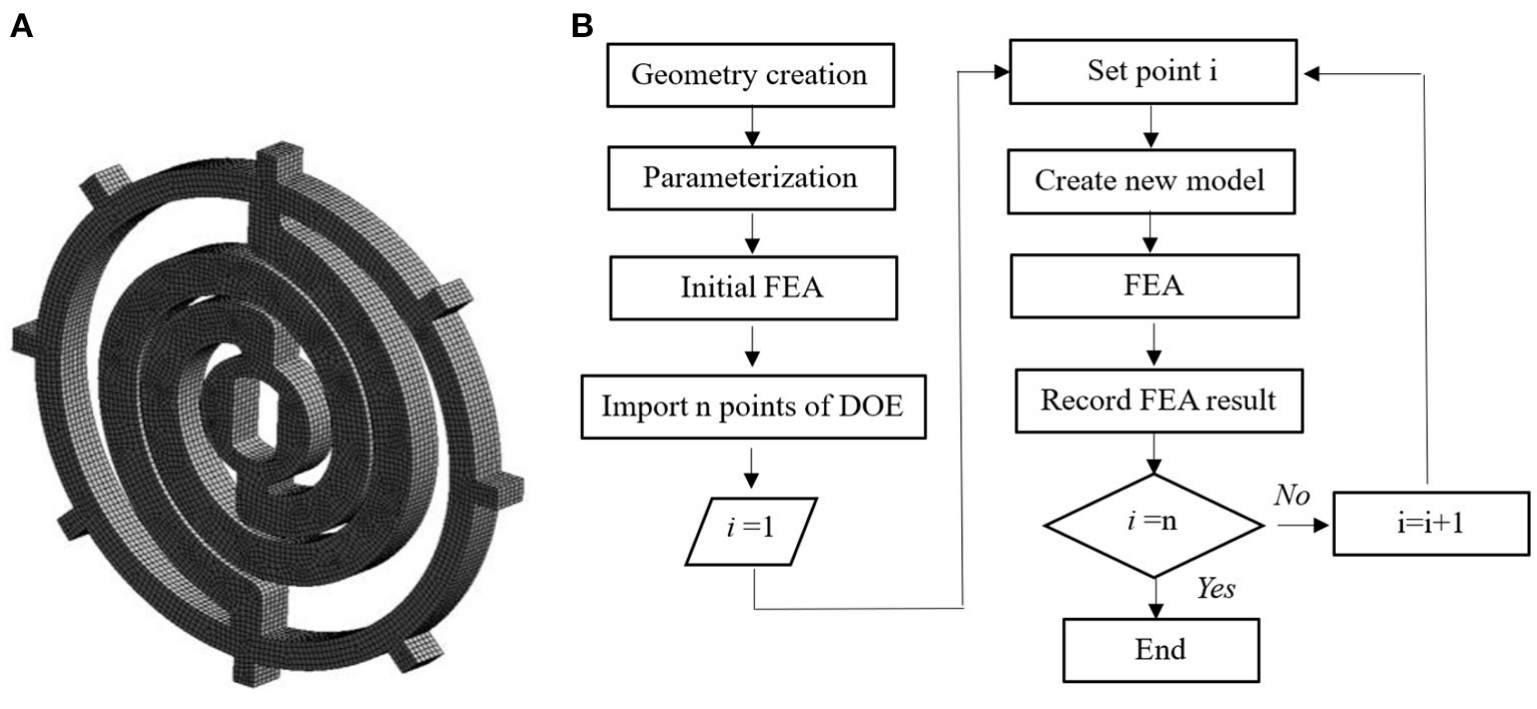
The mesh created and flow chart for the finite element analysis (FEA) of the flexible coupler. **(A)** Mesh created. **(B)** Flow chart.

The DOE method is used to investigate the effects of the geometrical parameters on the stiffness of the flexible coupler. The mesh created is shown in Figure 3A. Totally 32613 elements are generated. Eight lugs are created with equal space for anti-rotation on the outer diameter of the flexible coupler.

The beam thickness is from 6 to 8 mm with the step of 0.2 mm; the initial inner radium is from 22 to 30 mm with the step of 0.2 mm; the initial outer radium is from 34 to 41 mm with the step of 0.2 mm; the load applied is from 10 Nm to 60 Nm with the step of 1 Nm. The DOE flow chart is shown in Figure 3B.

**Figure 4:**
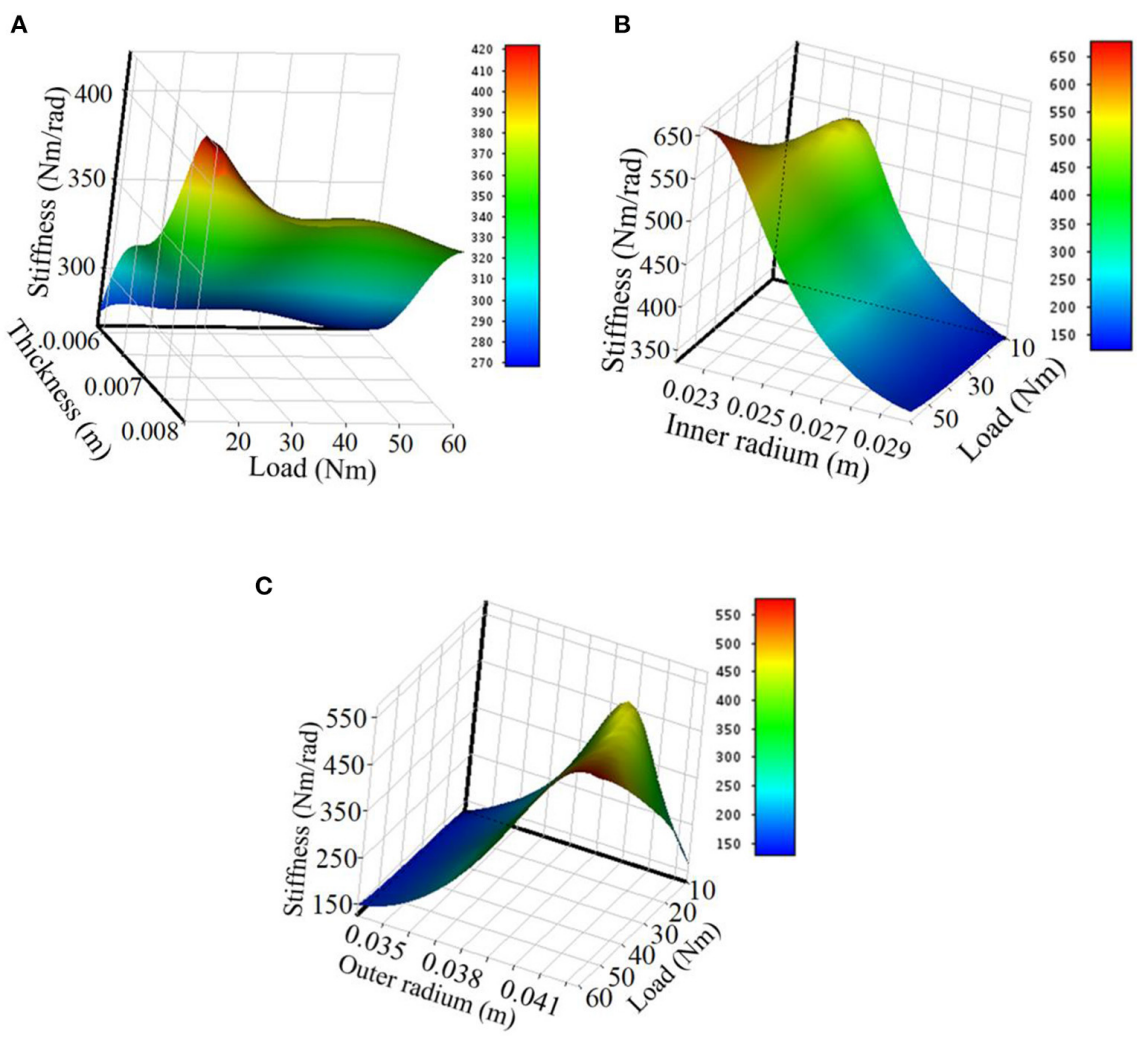
The effects of the geometrical parameters on the stiffness. **(A)** Thickness effects on the stiffness. **(B)** Inner radium effects on the stiffness. **(C)** Outer radium effects on the stiffness.

The surface response of the effects of the beam thickness and load is plotted as shown in Figure 4A. The stiffness increases with the beam thickness but not in a linear relation; it decreases with the load increase, quickly in the beginning and gently afterward.

The effects of the inner radium and load are plotted as shown in Figure 4B. The stiffness decreases with the inner radium increase and increases gently with the load.

The effects of the outer radium and load are shown in Figure 4C. The stiffness increases with the outer diameter if the load is big (50–60 Nm); the stiffness increases with the outer diameter in the beginning and then decreases if the load is low (10–20 Nm).

## 3. Optimization of the flexible coupler by neural network

According to the response relationship between the topological structure parameters and the mechanical properties of the flexible coupler obtained above (introduced in Section 2), the flexible coupler is optimized by the neural network and the DOE optimization method.

### 3.1. Neural network for stiffness modeling

The flexible coupler stiffness, *K*, is a function of the beam thickness, initial inner radium, initial outer radium, external load, and the properties of the given material (50CrVA) in Section 2.2.


(1) $$\begin{array}{l} K=f\left(E,\rho ,\gamma ,R_{1},R_{2},h,T\right) \end{array}$$

where *E* is the Young's modulus, ρ is the density, γ is the Poison's ratio and *T* is the torque.

According to the analysis of the parameter effects on the stiffness, the stiffness function is too complex to formalize. To create the stiffness function for further geometrical parameter optimization, the neural network method is a good option for complex function modeling (Wang et al., 2016).

**Figure 5:**
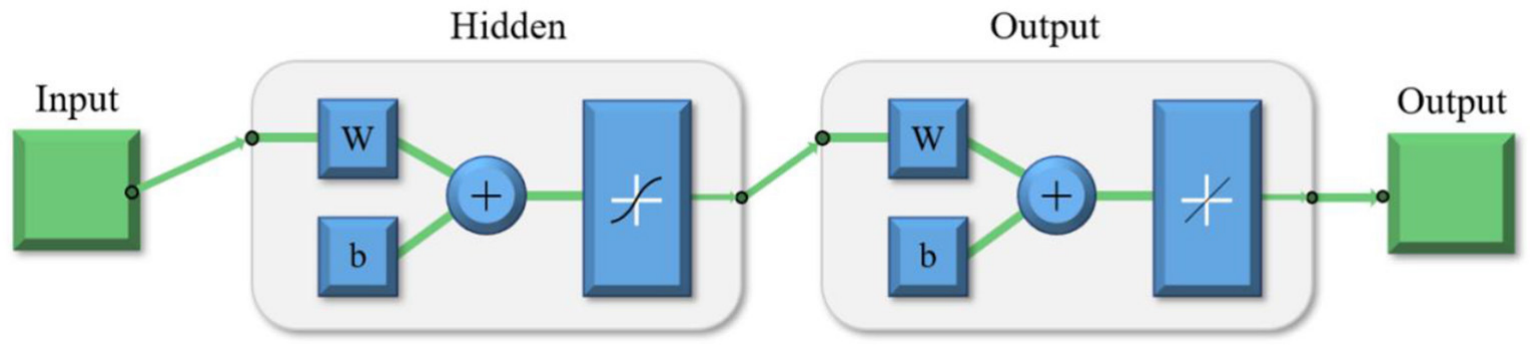
Neural network for the stiffness modeling.

The neural network is composed of the input, hidden layer, output layer, and output, as shown in Figure 5, where W is the weight and b is the bias.

The neural network output is the displacement angle, *A* and


(2) $$\begin{array}{l} A=\frac{T}{K}=\frac{T}{f\left(E,\rho ,\gamma ,R_{1},R_{2},h,T\right)}\left(rad\right). \end{array}$$

The Levenberg-Marquardt algorithm was used as the training algorithm of the neural network to train the neural network model, including the input layer, the hidden layer, and the output layer. In the hidden layer, 20 neurons are used for training with the Levenberg-Marquardt method (Huang and Ma, 2019). The finite element analysis results in DOE are used for the neural network training. 70% of the results are used for the training dataset, 15% are used for the calibration dataset and the rest 15% are used for the test dataset. The sample number and fitting mean square errors are listed in Table 3.

**Table 3:** Sample number and fitting mean square errors.

**Data**	**Training dataset**	**Test dataset**	**Calibration dataset**
Sample number	126	27	27
MSE	3.515 × 10^−9^	1.782 × 10^−8^	3.18 × 10^−7^

**Figure 6:**
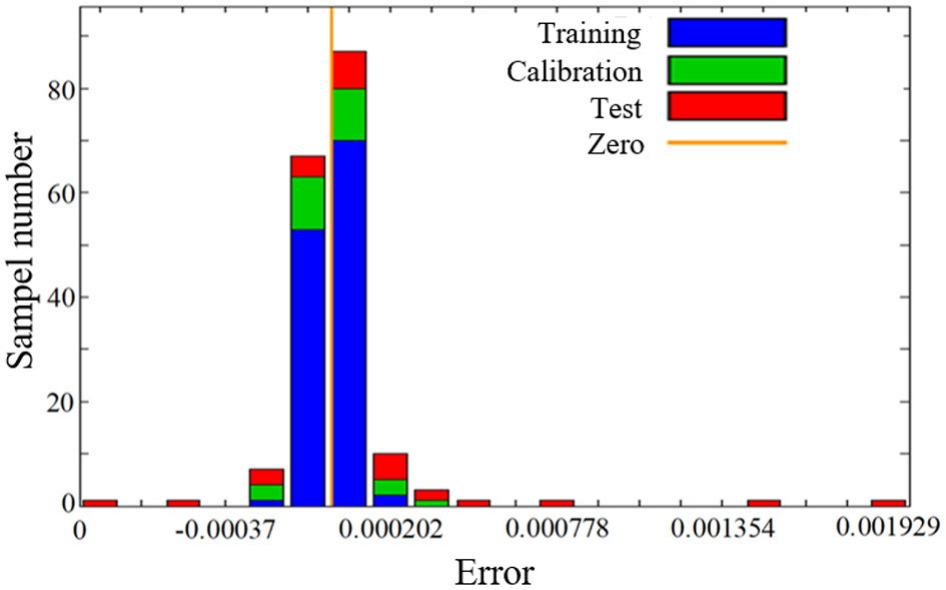
Error and sample distribution of the neural network.

The error and sample distribution are shown in Figure 6. Most of the samples concentrate around the error of 0. Error is the difference between the flexible coupler's target deformation angle and the flexible coupler's output deformation angle.

### 3.2. Geometrical parameter optimization with DOE

DOE method can be used for geometrical optimization, particularly for the parameterized topological structure. For the flexible coupler, three geometrical parameters are involved in the optimization, *R*_1_, *R*_2_, and *h*.

The objective of the geometrical optimization is to acquire the optimal parameters, *R*_1_, *R*_2_, and *h*, of the flexible coupler to ensure the stiffness is closer to the design requirement, 450 Nm/rad, with the error no more than 0.3% in the given load range, 0 Nm ≤*T* ≤ 60 Nm.

The above geometrical optimization objective can be summarized as the stiffness mean square error is the minimum in the load range of (10, 60) Nm with the step of 10 Nm, the objective function is defined as,


(3) $$\begin{array}{c} S=\frac{1}{6}\overset{6}{\underset{i=1}{\sum }}{\left(\frac{10i}{A\left(10i\right)}-450\right)}^{2} \end{array}$$

#### 3.2.1. Initial iteration

The initial iteration is to find the possible optimal point with a big step to ensure the computation load on an affordable level. The parameter ranges and steps are listed in Table 4. The beam thickness, *h*, is from 6 mm to 8 mm with a step of 1 mm; the initial inner radium is from 24 mm to 30 mm with a step of 2 mm; the initial outer radium is from 34 to 40 mm with a step of 2 mm. The load applied is from 10 Nm to 60 Nm with a step of 10 Nm. The iteration results show that the best point is the parameter set 17, with *h* = 7 mm, *R*_1_= 26 mm, and *R*_2_ = 40 mm. However, the average stiffness is only 387.76 Nm/rad, which is 14.78% lower than the target stiffness, 450 Nm/rad.

**Table 4:** Parameter ranges and steps of initial iteration.

**Parameter**	**Min (mm)**	**Max (mm)**	**Step**
*R_1_*	24	30	2
*R_2_*	34	40	2
*h*	6	8	1

#### 3.2.2. The second iteration

The result of the initial iteration shows the outer radium, *R*_2_, is 40 mm, which reaches the boundary. The second iteration is to reduce the parameter range according to the result of the initial iteration and refine the step to half level. Meanwhile, slightly increase the up limit of *R*_2_ to 41 mm. The parameter ranges and steps are listed in Table 5. The second iteration results show that the best result with regard to the objective function value is the geometrical parameter set 1, (*h* = 6.5 mm, *R*_1_ = 25 mm, *R*_2_ = 39 mm), with the objective function value of 6.6 (Nm/rad)^2^. The second best is the geometrical parameter set 13 (*h* = 7 mm, *R*_1_ = 27 mm, *R*_2_ = 41 mm), with the objective function value of 65.5 (Nm/rad)^2^. Both the best and second-best points are selected for further iteration to ensure the real global optimal point to be found.

**Table 5:** Parameter ranges and steps for the second iteration.

**Parameter**	**Min (mm)**	**Max (mm)**	**Step**
*R_1_*	25	27	1
*R_2_*	39	41	1
*h*	6.5	7.5	0.5

#### 3.2.3. The third iteration

The third iteration is around the best (Point 1) and the second-best point (Point 2) obtained in the second iteration with a refined step of 25% of the initial step used in the initial iteration. The parameter ranges and steps for Point 1 are listed in Table 6, and those for Point 2 are listed in Table 7. The third iteration results of Point 1 show that the best point is the geometrical parameter set 14 (*h* = 6 mm, *R*_1_ = 25 mm, *R*_2_ = 39.5 mm) with the objective function value of 4.2 (Nm/rad)^2^. The third iteration results of Point 2 show that the best point is the geometrical parameter set 18 (*h* = 7 mm, *R*_1_ = 27.5 mm, *R*_2_ = 41.5 mm) with the objective function value of 6.6 (Nm/rad)^2^.

**Table 6:** Parameter ranges and steps for the third iteration of Point 1.

**Parameter**	**Min (mm)**	**Max (mm)**	**Step**
*R_1_*	24.5	25.5	0.5
*R_2_*	38.5	39.5	0.5
*h*	6	7	0.5

**Table 7:** Parameter ranges and steps for the third iteration of Point 2.

**Parameter**	**Min (mm)**	**Max (mm)**	**Step**
*R_1_*	26.5	27.5	0.5
*R_2_*	40.5	41.5	0.5
*h*	6.5	7.5	0.5

The comparison of the third iteration results about Point 1 and Point 2 shows that the optimal geometrical parameter set is (*h* = 6 mm, *R*_1_ = 25 mm, *R*_2_ = 39.5 mm).

**Figure 7:**
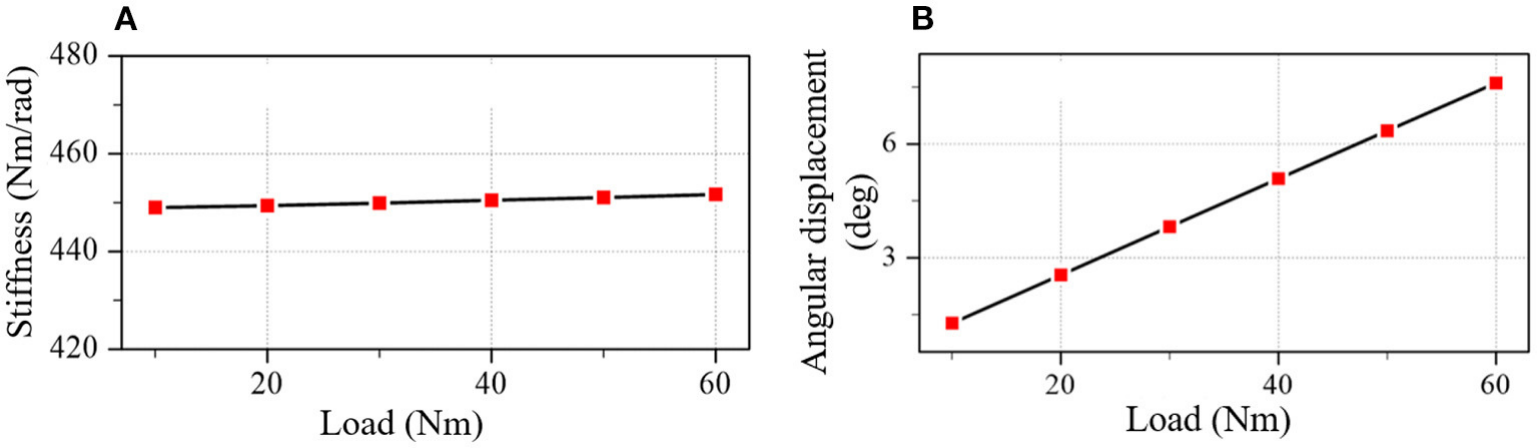
The stiffness and angular displacement of the optimal coupler. **(A)** Stiffness. **(B)** Angular displacement.

The result shows that the parameter-optimized coupler meets the optimization target. The stiffness and angular displacement of the optimal coupler are plotted in Figure 7. The stiffness means the square error is 0.91 (Nm/rad)^2^ and the stiffness error is less than 0.2% of the target stiffness (450 Nm/rad).

### 3.3. Strain stress analysis of the optimal coupler

**Figure 8:**
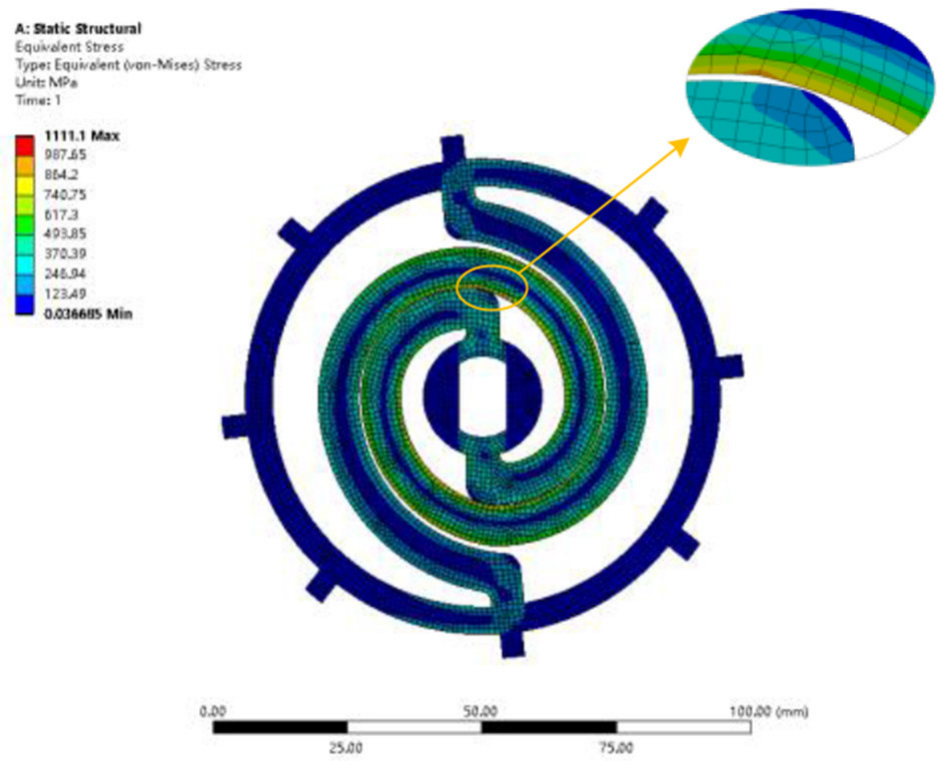
Static structure analysis of the optimal coupler.

The optimal coupler properties were verified by FEA. The optimal coupler is analyzed with FEA under the load of 60 Nm applied on the central hole. The stress distribution is shown in Figure 8. The maximum stress is 1111.1 MPa, which does not exceed the yield strength of 50CRVA (1127 MPa).

## 4. Experiment and result

To verify the optimization result, the flexible coupler was processed according to the optimized parameters, and a test rig was built for experimenting.

### 4.1. The setup of experiment

**Figure 9:**
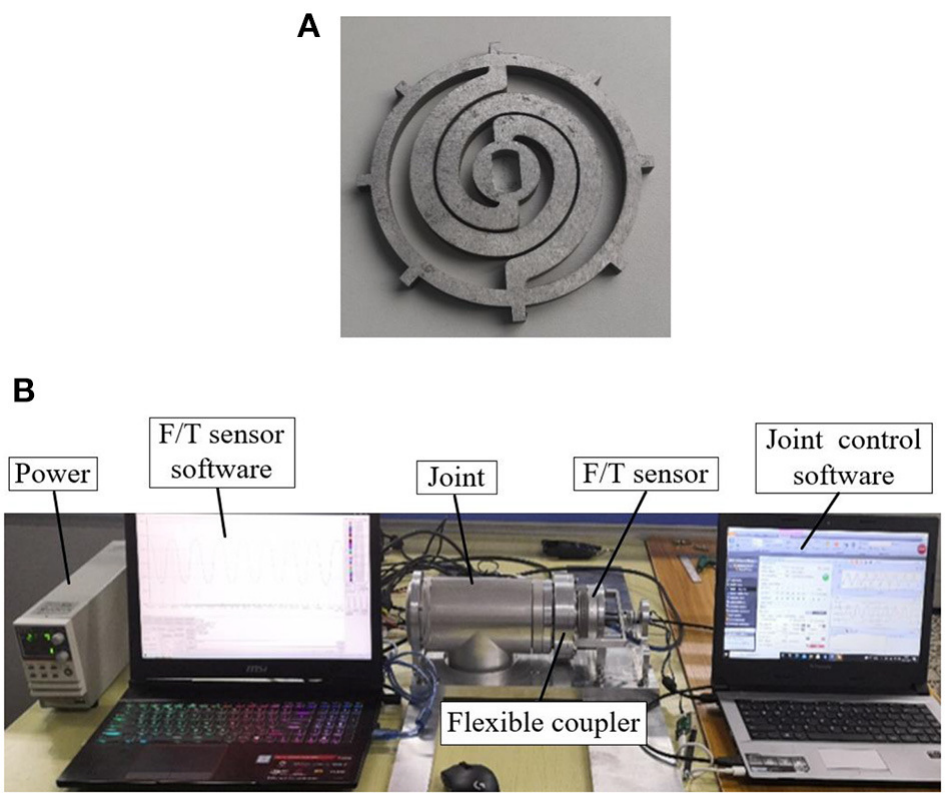
The setup of experiment. **(A)** The optimal flexible coupler. **(B)** Test rig for the flexible coupler.

The test piece of the optimal coupler is fabricated with a wire EDM process, as shown in Figure 9A. The material is 50 CrVA. The test rig is shown in Figure 9B. The joint equipped with a flexible coupler is fixed on the base of the test rig. One end of the F/T sensor (SRI-M4325K1) is connected to the output end of the flexible coupler through a flange, and the other end is fixed on the base. The work of the joint is controlled by the motor driver (ELMO G-SOLTWI15/100EE1) and the software (EAS II). The angular displacement is measured by the encoder (RLS MRA029BC010DSE00) on the opposite of the flange. Output force and moment of the flexible coupler are measured by the F/T sensor, and the data is recorded by software (iDAS R&D). Detailed information on the F/T sensor is shown in Table 8.

**Table 8:** The information of F/T sensor.

**Parameter**	**Value**	**Unit**
Sensor	SRI-M4325k1	/
Fx	200	N
Fy	200	N
Fz	400	N
Mx	20	Nm
My	20	Nm
Mz	20	Nm
Crosstalk	2.5	%F. S
Non-linearity	0.5	%F. S
Hysteresis	0.5	%F. S
Free air resonant freq	1,500	Hz

### 4.2. Experiment steps

#### 4.2.1. Step 1: Gage calibration

The F/T sensor and encoder must be calibrated before the experiment. The calibration of the F/T sensor adopts the standard weight loading method, and the calibration of the encoder adopts the standard protractor measurement.

#### 4.2.2. Step 2: Equipment operation

Power on all experiment equipment (motor, F/T sensor, driver, and so on) and start the software to ensure that all equipment is functional. Rotate the gearbox (shown in Figure 1) output shaft to the initial point (the point where the deformation of the flexible coupler is 0). Reset the indications of the F/T sensor and encoder to zero.

#### 4.2.3. Step 3: Experiment and data record

Control the output shaft of the gearbox (shown in Figure 1) by the software to rotate to the experiment point and stay for 3 s, record the F/T sensor data of the 3 s, and take the average value as the measurement result. After one test, return the output shaft position of the gear box to the initial point (introduced in Section 4.2.2), reset the indications of the F/T sensor and encoder to zero. Then start the next measurement. Each group of data is measured three times, and the average value is taken as the experiment result.

### 4.3. Experiment result

**Figure 10:**
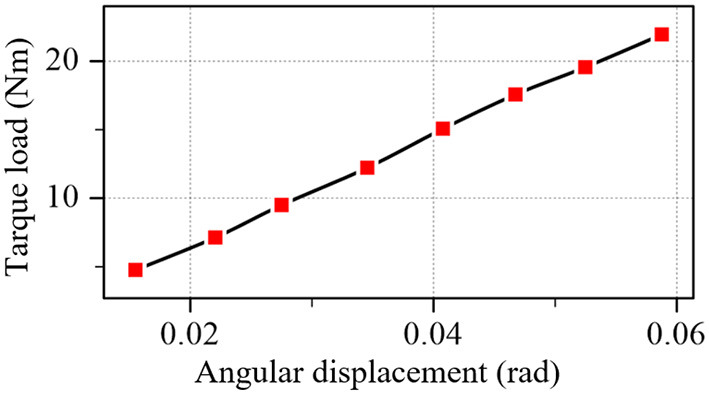
Test results of the optimal coupler.

The experiment result of the optimal flexible coupler is plotted in Figure 10. The stiffness error is within the range of ±0.25 % and align with the optimization target (introduced in Section 3.2, the error is no more than 0.3%).

## 5. Conclusion

This paper proposed a neural network aided flexible joint structure optimization method with DOE for the nuclear power plant inspection robot. With this method, the flexible coupler of the joint was optimized with regard to the minimum mean square error of the stiffness. The optimal flexible coupler was demonstrated and tested. According to the analysis and test results, the following conclusions can be drawn:

(1) The neural network method can be used for modeling the stiffness of the flexible coupler with regard to the geometrical parameters as well as the load on the base of the results of the FEA with DOE.
(2) With the aid of the neural network model of the stiffness, the dual-spiral flexible coupler structure can be fully optimized for a target stiffness, 450 Nm/rad in this case, and a given error level, 0.3% in the current case, with regard of the different loads.
(3) The optimization with DOE should be iterated at least three times with the refined parameter ranges and steps to ensure the result is the real global optimal.
(4) The optimal flexible coupler meets the design requirement; the stiffness is 450 Nm/rad, and the error is <0.3%.

Further research includes (a) the modeling and analysis of the dynamic performance of the flexible joint; (b) the control system and frequency response analysis of the flexible joint; (c) the other tests of the inspection robot.

## Data availability statement

The original contributions presented in the study are included in the article/supplementary material, further inquiries can be directed to the corresponding author.

## Author contributions

GW and XC: conceptualization, writing-review and editing, and funding acquisition. JL: methodology. XM, JW, SH, BP, and RT: writing-original draft preparation. GW, JL, and XC: significance contributions to the manuscript. All authors read and agreed to the published version of the manuscript.
